# Quantitative and qualitative analyses of human salivary NEFA with gas-chromatography and mass spectrometry

**DOI:** 10.3389/fphys.2012.00328

**Published:** 2012-08-16

**Authors:** Bhushan V. Kulkarni, Karl V. Wood, Richard D. Mattes

**Affiliations:** ^1^Department of Nutrition Science, Purdue UniversityWest Lafayette, IN, USA; ^2^Department of Chemistry, Purdue UniversityWest Lafayette, IN, USA

**Keywords:** NEFA, gas chromatography–mass spectrometry, salivary, human

## Abstract

Salivary non-esterified fatty acids (NEFA) are proposed to play a role in oral health, oral fat detection, and they may hold diagnostic and prognostic potential. Yet, little is known about the array and concentrations of NEFA in saliva. The aim of the study was to conduct qualitative and quantitative analyses of salivary NEFA in healthy humans and to present a new, efficient protocol to perform such analyses. Resting saliva samples from fifteen participants were collected. The salivary lipids were extracted using a modified Folch extraction. The NEFA in the extracted lipids were selectively subjected to pentafluorobenzyl bromide (PFB) derivatization and qualitatively and quantitatively analyzed using gas chromatography–mass spectrometry (GC–MS). A total of 16 NEFA were identified in resting saliva. The four major NEFA were palmitic, linoleic, oleic, and stearic acids. Their concentrations ranged from 2 to 9 μM. This is the first study to characterize individual human salivary NEFA and their respective concentrations. The method used in the study is sensitive, precise, and accurate. It is specific to fatty acids in non-esterified form and hence enables analysis of NEFA without their separation from other lipid classes. Thus, it saves time, reagents and prevents loss of sample. These properties make it suitable for large scale analysis of salivary NEFA.

## Introduction

Non-esterified fatty acids (NEFA) play an important role in human metabolism. They act as a source of energy via β oxidation, as building blocks for synthesis of phospholipids, lipoproteins, and glycolipids, and as intra- and intercellular signaling molecules. More specifically, they are secreted in human saliva (Larsson et al., 1996) where they may serve multiple roles. They likely contribute to the maintenance of oral health (Slomiany et al., 1985), signal the presence of fats in the oral cavity (Mattes, 2009a) and could hold diagnostic and prognostic information (Higashi et al., 2008; Izawa et al., 2012).

Stimulated human salivary NEFA concentrations are approximately 1 μg/mL (Larsson et al., 1996). Concentrations are elevated in certain diseases such as cystic fibrosis and Sjogren's syndrome and decreased in others such as parotitis (Slomiany et al., 1985). They are also higher in caries susceptible (Slomiany et al., 1982a; Tomita et al., 2008), and high calculus forming (Slomiany et al., 1980) adults than those less affected by these processes. The basis of the association between salivary NEFA concentrations and general oral health is not well characterized. NEFA may only serve as markers or play a causal role. In either case, better knowledge of individual fatty acid contributions should improve the sensitivity and specificity of the associations. For example, individual NEFA such as lauric, linoleic, and oleic acids suppress plaque formation and dissolution of hydroxyapatite at concentrations between 31 and 1000 μg/mL (Schuster et al., 1980). However, such concentrations are generally not present in saliva (Larsson et al., 1996) raising questions about their health effects. Better knowledge of individual NEFA concentrations may aid in determining their utility in disease etiology, diagnosis, and management.

In the oral cavity, humans can detect NEFA varying in chain length and saturation in the absence of textural, olfactory, and visual cues (Chalé-Rush et al., 2007; Mattes, 2009a). The minimum concentration of a NEFA in saliva that can be detected orally is termed its detection threshold. Humans can also differentiate and rank order suprathreshold concentrations of NEFA when non-gustatory cues are masked (Mattes, 2009b). Thus, the evidence suggests the presence of a gustatory NEFA signaling system in the human oral cavity. This gustatory NEFA signaling has been proposed to play a role in the oral detection of fat (Mattes, 2011). However, fats in the human diet are present in predominantly triglyceride form and human lingual lipase, the enzyme required for generating NEFA from dietary fats, has very weak activity (Stewart et al., 2010). Knowledge of which NEFA are present in saliva during oral processing of high-fat food and whether their salivary concentrations are higher than their respective detection thresholds is crucial in understanding whether NEFAs can act as a signal for the presence of fat in oral cavity. Thus, qualitative and quantitative analyses of salivary NEFA achieved during oral processing of fatty foods are important.

Assessment of dietary intake is crucial for nutrition research. Currently available methods for estimation of dietary intake such as 24 h recalls, food records, and food frequency questionnaires are based on self-reporting and have well-documented weaknesses such as under-reporting (Rennie et al., 2007). An objective analytical method should help to accurately assess dietary intake. The salivary fatty acid composition reflects the fatty acid composition of the diet in primates and humans (Alam and Alam, 1982; Actis et al., 2005) and thus analysis of salivary NEFA may provide an objective and non-invasive method for dietary fat intake assessment.

A number of studies on salivary lipids have analyzed the total quantity of salivary NEFA and fatty acid composition of other salivary lipids such as triacylglycerol, diacylglycerol, monoacylgycerols, and phospholipids in which fatty acids are present in esterified forms (Mandel and Eisenstein, 1969; Rabinowitz and Shannon, 1975; Slomiany et al., 1980, 1982a,b, 1983; Larsson et al., 1996; Actis et al., 2005; Tomita et al., 2008). However, there has been no study of the composition and concentrations of salivary fatty acids that are in the non-esterified form. This is due, in part, to the lack of a suitable method to analyze these compounds. The following protocol was followed to provide both a quantitative as well as qualitative analyses of salivary NEFA.

## Materials and method

### Saliva collection

Fifteen 18–50-year-old, self-reported, non-smoking individuals (4 males, 11 females) with self-described good overall and oral health as well as normal weight (Body Mass Index = 18.5–25 kg/m^2^) were recruited. The participants were instructed not to drink or eat anything except water and refrain from use of any oral hygiene products for 2 h before their study visit. Ten to twelve milliliters of whole resting (no mechanical or chemical stimulation) saliva was collected in a pre-chilled, sterile, disposable 15 mL centrifuge tubes (VWR, Randor, PA). The tubes were chilled in ice during collection of the saliva.

### Extraction of lipids

The saliva samples were immediately centrifuged at 4500 rpm for 30 min at 4°C using an Allegra 25 R centrifuge (Beckman Coulter, Brea, CA). The clear supernatant was transferred to a 50 mL round-bottom pyrex centrifuge tube (Corning Inc, Corning, NY). Nonadecanoic acid (Nu-Chek Prep, Elysian, MN) was added as an internal standard to the supernatant in a volume dependent amount so as to achieve a 10 μM final concentration. The supernatant was lyophilized over 8–10 h using a freeze-dry system (Labconco, Kansas City, MO). Next, 0.5 mL of deionized water was added to the lyophilized samples. The lipids were extracted from the rehydrated samples with chloroform and methanol (11 and 5.5 mL, respectively) using a modified Folch extraction method (Folch et al., 1957). The extraction led to the formation of a biphasic liquid system. The upper phase was discarded. The lower phase containing the extracted lipids was transferred to a capped glass tube and dried under nitrogen.

### Derivatization

Selective pentafluorobenzyl bromide (PFB) derivatization of NEFA present in the extracted lipid was achieved (Quehenberger et al., 2008) by adding 250 μL of each of a 1% solution of diisopropylethylamine in acetonitrile and a 1% solution of PFB in acetonitrile to the extracted lipids. The reaction mixture was allowed to sit for 30 min at room temperature. The samples were dried under nitrogen, dissolved in 50 μL of acetonitrile and transferred to gas chromatography (GC) vials with an adapter (ThermoFisher Scientific, Waltham, MA).

### GC–MS analysis

The PFB derivatives of NEFA were separated by GC (Agilent, 7890A; Agilent Tech, Santa Clara, CA) using a HP-5 MS, 30 m × 0.25 mm capillary column. The Helium carrier gas flow rate was 1.2 mL/min. The oven temperature was set at 150°C for 0.1 min prior to being ramped to 320°C at 10°C/min for GC separation. The PFB derivatives of NEFA were identified and quantified after their separation by GC, using an Agilent 5975c Mass Spectrometer (MS). (M-H)^−^ ions of NEFA PFB derivatives were obtained using negative ion chemical ionization with methane as the reagent gas and source temp of 150°C (Quehenberger et al., 2008). The MS scan range was of 42–500 m/z. The GC–MS output was analyzed using Wsearch32 (www.wsearch.com.au Ver 1.6.2005) software.

All the chemicals used in the protocol were “ACS reagent” grade and were obtained from Sigma-Aldrich, (St. Louis, MO) unless stated otherwise. The study protocol was approved by University Institutional Review Board. A written informed consent was obtained from all participants prior to their enrollment in study.

### Calculation of NEFA concentrations

Concentrations of NEFA in the biological test sample are calculated by using nonadecanoic acid as in internal standard. The internal standard was added to the salivary supernatant before the lipids were extracted from the supernatant.

A standard chloroform solution containing equimolar concentrations of NEFA that were detected in the biological samples and the nonadecanoic acid (internal standard) were analyzed in triplicate by GC–MS. The absolute concentration of a NEFA corresponds to the absolute peak area of the NEFA in the chromatogram. The average ratio of absolute peak area of a NEFA to that of the nonadecanoic acid in the chromatogram of standard solutions was calculated to derive a correction factor for each NEFA. The correction factor for a NEFA (*C*), the absolute peak areas of the NEFA (*A*1), and nonadecanoic acid (*A*2) in the chromatogram of a biological test sample and the known concentration of nonadecanoic acid as an internal standard in the biological sample (10 μM) were used to calculate the absolute micromolar concentration of the NEFA (*M*) in the biological test sample using following formula:

$$M=(A1/A2)C^{*}10$$

where,

*M* = absolute micromolar concentration of a NEFA in test sample
*A*1 = absolute peak area of the NEFA in test sample
*A*2 = absolute peak area of nonadecanoic acid in test sample
*C* = correction factor for the NEFA

### Test for derivatization selectivity for NEFA

A mixture of 0.009 mg of tripalmitin (a triacylglycerol of palmitic acid), 0.003 mg of Dipalmityl and 0.003 mg of monopalmityl glycerol (Di and monoacyl glycerol of palmitic acid), 0.012 mg and 0.002 mg of cholesterol ester and phospholipid of palmitic acid, respectively, and 0.006 mg of myristic acid was derivatized using PFB derivatization followed by the GC–MS method described above. If PFB derivatization is specific for NEFA, only the peak for the myristic acid derivative will be seen in the output mass chromatogram as it is in non-esterified form and no peak should be seen for palmitic acid as it is present in esterified forms.

### Linearity and accuracy

Three standard solutions in chloroform containing mixtures of 40 μL of a 1 mM solution of nonadecanoic acid (internal standard) with 20, 40, and 80 μL of a 1 mM solution of stearic acid were derivatized and quantified by GC–MS using the above protocol. The known concentrations of stearic acid in the three standard solutions were compared against the concentrations calculated from the GC–MS output to determine the linearity and accuracy of the method.

## Results

**Figure 1:**
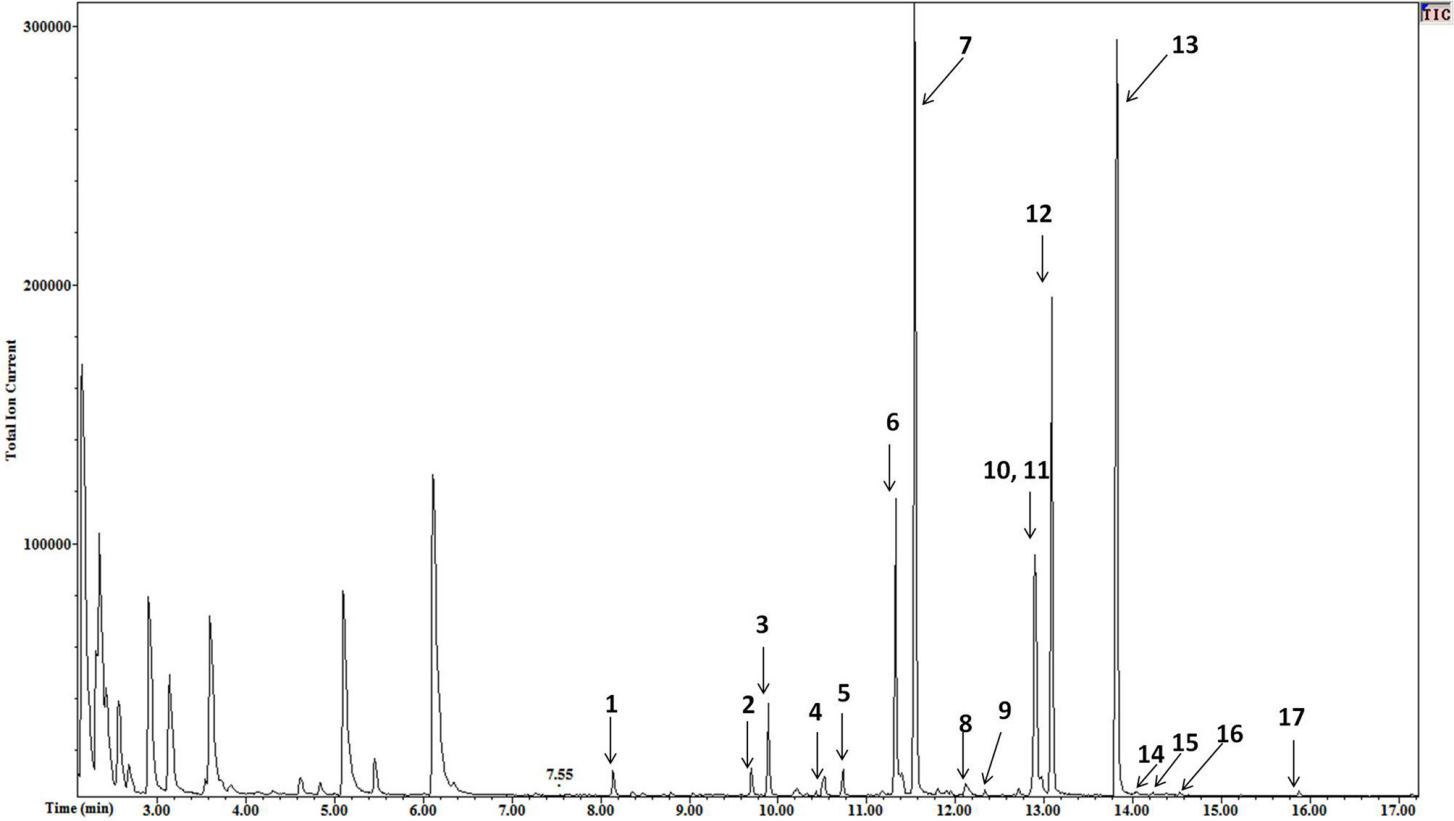
**Mass chromatogram of resting saliva**. The peaks of NEFA derivatives are numbered as follows 1 = lauric, 2 = myristoleic, 3 = myristic, 4 = pentadecenoic, 5 = pentadecanoic, 6 = palmitoleic, 7 = palmitic, 8 = heptadecaenoic, 9 = margaric, 10 = linoleic, 11 = oleic, 12 = stearic, 13 = Nonadecanoic (internal standard), 14 = arachidonic, 15 = eicosatrienoic, 16 = arachidic, and 17 = behenic acid. High resolution provided adequate separation of peaks. High sensitivity enabled a high signal to noise ratio varying from 30 to 100. A minimum signal to noise ratio of 10 was used to define a peak.

The high resolution of the gas chromatograms enabled adequate separation of peaks and identification of each NEFA derivative (Figure 1). The PFB derivatives of NEFA were analyzed using negative ion chemical ionization, producing (M-H)^−^ anions. The mass to charge ratio and retention times of detected (M-H)^−^ anions of NEFA PFB derivatives are given in Table 1. Palmitic (16:0), oleic (18:1), linoleic (18:2), and stearic (18:0) acids were the four major NEFA identified in resting saliva (Table 2). Their individual concentrations ranged from 2 to 9 μM (approximately 0.5 to 2.5 μg/mL). In addition to these four major NEFA, twelve other NEFA were detected at concentrations less than 1 μM including lauric acid (12:0), myristic acid (14:0), myristoleic acid (14:1), pentadecanoic acid (15:0), pentadecenoic acid (15:1), palmitoleic acid (16:1), margaric acid (17:0), heptadecenoic acid (17:1), arachidic acid (20:0), eicosatrienoic acid (20:3), arachidonic acid (20:4), and behenic acid (22:0).

**Table 1:** **GC–MS analysis of pentafluorobenzyl bromide derivatives of NEFA**.

**NEFA**	**Retention time (min)**	**Mass to charge ratio [M-H]^−^*m/z***
Lauric acid (12:0)	8.14	199
Myristic acid (14:0)	9.90	227
Myristoleic acid (14:1)	9.72	225
Pentadecanoic acid (15:0)	10.74	241
Pentadecenoic acid (15:1)	10.52	239
Palmitic acid (16:0)	11.57	255
Palmitoleic acid (16:1)	11.33	253
Margaric acid (17:0)	12.34	269
Heptadecenoic acid (17:1)	12.14	267
Stearic acid (18:0)	13.11	283
Oleic acid (18:1)	12.93	281
Linoleic acid (18:2)	12.93	279
Nonadecanoic acid (19:0)	13.84	297
Arachidic acid (20:0)	14.55	311
Eicosatrienoic acid (20:3)	14.24	305
Arachidonic acid (20:4)	14.05	303
Behenic acid (22:0)	15.89	339

**Table 2:** **Major salivary NEFA (number of participants = 15)**.

**Salivary NEFA**	**Concentrations (μM mean ± SEM)**
Palmitic acid	8.21 ± 1.09
Oleic acid	2.09 ± 0.61
Linoleic acid	1.61 ± 0.56
Stearic acid	6.57 ± 0.46

**Figure 2:**
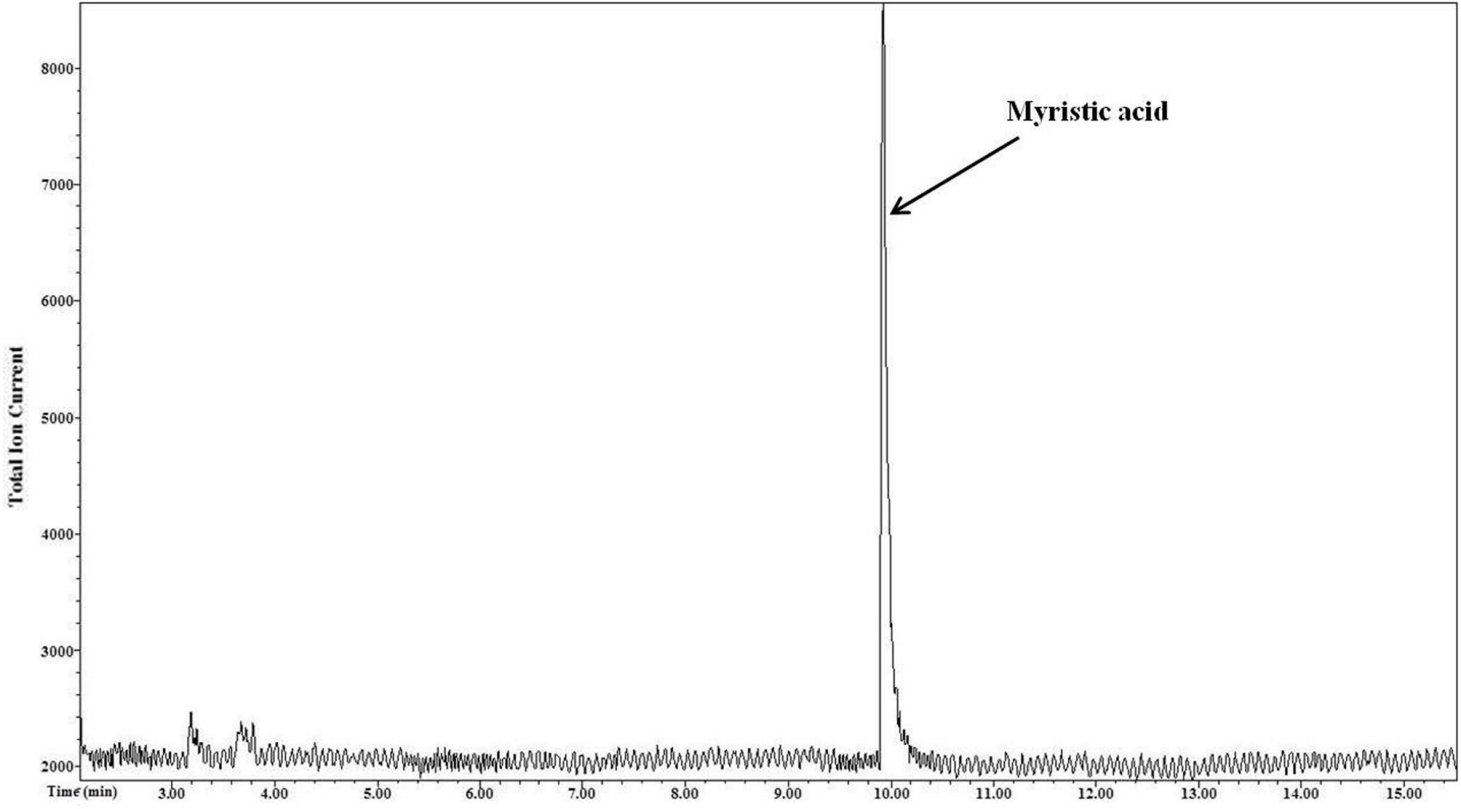
**Selective derivatization of NEFA**. Myristic acid that was in a non-esterified form underwent derivatization and only the peak of its derivative is seen in the resultant mass chromatogram. Esterified palmitic acid that was present in other lipid classes tri, di, and monoacylgycerols, phospholipids, and cholesterol esters was not derivatized.

The output mass chromatogram (Figure 2) of the test of selectivity of derivatization showed only the myristic acid peak (present in non-esterified form) and no peak for palmitic acid (present in esterified form) proving selectivity of the derivatization method for NEFA.

**Figure 3:**
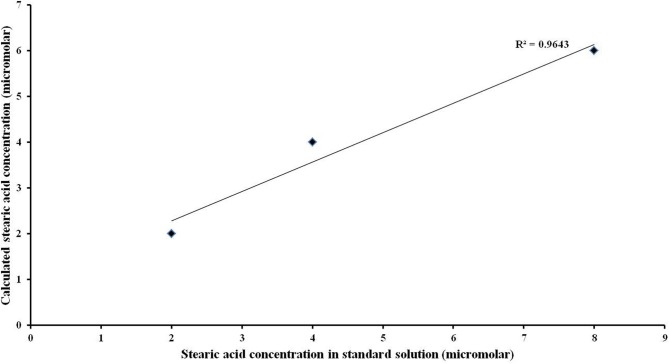
**Linearity of the quantitative analysis**. High linear correlation was observed between actual values of stearic acid concentration and its corresponding calculated values.

The correlation between the known stearic acid concentration and the estimated stearic acid concentration in a standard solution (R^2^) was 0.9643 (Figure 3).

## Discussion

This is the first reported qualitative and quantitative analyses of individual salivary NEFA. Sixteen different NEFA were detected in saliva. Palmitic acid had the highest mean concentration followed by stearic, oleic, and linoleic acids, respectively. The other 12 NEFA were present at concentrations of less than 1 μM. The four major NEFA found in saliva are also the predominant fatty acids present in other salivary lipid classes such as glycerides, cholesterol esters, phospholipids, and glycerolipids (Tomita et al., 2008). They are the most common fatty acids in the human diet as well (USDA database). Dietary total fatty acid composition is known to influence total salivary fatty acid composition and the result of this study suggests dietary fatty acid composition may also influence salivary NEFA composition. Measurement of salivary NEFA following dietary interventions employing different quantities and varieties of NEFA will be required to assess this hypothesis.

### Development of protocol

#### Extraction of lipids

A modified Folch (Folch et al., 1957) method used in our protocol is commonly used to extract lipids from saliva (Mandel and Eisenstein, 1969; Rabinowitz and Shannon, 1975; Slomiany et al., 1980, 1982a,b, 1983; Larsson et al., 1996; Actis et al., 2005; Tomita et al., 2008).

#### Derivatization of NEFA

The commonly used approach to analyze NEFA in biological samples consists of extraction of total lipids by organic solvents, separation of extracted lipids into different lipid classes using either thin layer chromatography or solid phase extraction and subsequent derivatization of NEFA before analysis by gas /liquid chromatography and MS (Ruiz-Rodriguez et al., 2010). The step of separation of lipid classes is necessary since traditional derivatization methods such as methyl esterification are not specific to NEFA. The PFB derivatization introduced by Kawahara (1968) is specific to only NEFA and does not derivatize fatty acids in esterified form. PFB derivatization thus allows direct derivatization of NEFA after extraction of lipids from saliva without the prior separation of NEFA from other lipid classes using separation methods such as thin layer chromatography or solid phase extraction. This improvement saves time, reagents and prevents loss of sample caused by the extra separation step. PFB derivatization also has better sensitivity during MS analysis as compared to methyl esterification (Kawahara, 1968). Therefore after extracting lipids with the modified Folch extraction, NEFA were derivatized using PFB derivatization.

#### GC–MS analysis

PFB derivatives of salivary NEFA were analyzed by GC–MS in negative ionization mode using a protocol adapted from a method published by Quehenberger et al. (2008) to analyze multiple essential NEFA in macrophages.

### Characteristics of the protocol

#### Sensitivity

The protocol described in this paper has a sensitivity that enables detection of NEFA in saliva with a concentration as low as 1 nM as standard solutions containing NEFA at 1 nM concentration produced peaks with a signal to noise ratio higher than 10 (criteria used to define a peak in the chromatogram). This high sensitivity allowed detection of four major NEFA present at 2–9 μM concentrations (three orders of magnitude higher than nanomolar concentrations) as well as NEFA present at trace concentrations. The high sensitivity also enabled the analysis of individual saliva samples without the need for pooling multiple samples.

#### Selectivity

The salivary lipids consist of triacylglycerol, diacylglycerol, monoacylglycerol, cholesteryl ester, phospholipid, and NEFA. All these lipid classes were mixed in a proportion similar to that reported for salivary lipids (Larsson et al., 1996) to confirm the selectivity of the PFB derivatization. There were only two fatty acids, myristic acid, and palmitic acid, in the mixture. Myristic acid was present in a non-esterified form representing the NEFA lipid class while palmitic acid was present in an esterified form in all other lipid classes of the mixture. Unlike commonly used NEFA derivatization methods for analysis, such as methyl esterification, the PFB derivatization was confirmed to be specific to NEFA (myristic acid) as it derivatized only myristic acid whose peak was seen in the output chromatogram. The method did not derivatize the esterified fatty acid (palmitic acid) present in other salivary lipid classes such as glycerides, phospholipids, and cholesterol esters. Hence, there was no peak for palmitic acid in the chromatogram (Figure 2).

#### Precision

The protocol has high precision (relative standard deviation of 1.60, 6.31, 6.79, and 1.34 for palmitic, linoleic, oleic, and stearic acid, respectively) making it highly reproducible.

#### Linearity

The protocol has a high linear correlation (Figure 3) between actual NEFA concentration ranging from 2 to 8 μM and the corresponding calculated values, suggesting high accuracy. The concentration range of 2 to 8 μM was used for the analysis as the salivary NEFA concentration reported in this study falls within this range. Thus, this approach ensured complete conversion of NEFA in resting saliva.

## Conclusion

The method described in this paper is very sensitive, specific, precise, and accurate. Further, it is less cumbersome as there is no need for separation procedures and it enables qualitative as well as quantitative analyses of salivary NEFA. Therefore, it is suitable for saliva sample processing for research settings and diagnostic and prognostic purposes in clinical settings.

### Conflict of interest statement

The authors declare that the research was conducted in the absence of any commercial or financial relationships that could be construed as a potential conflict of interest.
